# Anticancer Activities of C_18_-, C_19_-, C_20_-, and Bis-Diterpenoid Alkaloids Derived from Genus *Aconitum*

**DOI:** 10.3390/molecules22020267

**Published:** 2017-02-13

**Authors:** Meng-Yue Ren, Qing-Tian Yu, Chun-Yu Shi, Jia-Bo Luo

**Affiliations:** 1 School of Traditional Chinese Medicine, Southern Medical University, Guangzhou 510515, China; Rmy0711@163.com (M.-Y.R.); dkdklm@163.com (Q.-T.Y.); 15626452676@163.com (C.-Y.S.); 2 Guangdong Provincial Key Laboratory of Chinese Medicine Pharmaceutics, Southern Medical University, Guangzhou 510515, China

**Keywords:** anticancer, genus *Aconitum*, diterpenoid alkaloids

## Abstract

Cancer is one of the most common lethal diseases, and natural products have been extensively studied as anticancer agents considering their availability, low toxicity, and economic affordability. Plants belonging to the genus *Aconitum* have been widely used medically in many Asian countries since ancient times. These plants have been proven effective for treating several types of cancer, such as lung, stomach, and liver cancers. The main effective components of *Aconitum* plants are diterpenoid alkaloids—which are divided into C_18_-, C_19_-, C_20_-, and bis-diterpenoid alkaloids—are reportedly some of the most promising, naturally abundant compounds for treating cancer. This review focuses on the progress of diterpenoid alkaloids with different structures derived from *Aconitum* plants and some of their derivatives with potential anticancer activities. We hope that this work can serve as a reference for further developing *Aconitum* diterpenoid alkaloids as anticancer agents.

## 1. Introduction

Cancer is one of the most common lethal diseases, with approximately 14 million new cases of cancer diagnosed and 8 million cancer-related deaths in 2012. This disease affects all populations in all regions according to the World Health Organization [1]. The five most common incident sites of cancers are the lung, breast, colorectum, prostate, and stomach, constituting half of incident sites worldwide [1]. In recent years, natural products, including materials originating from plants, animals and their derivatives, have been extensively studied as anticancer agents considering their availability, low toxicity, and economic affordability. Over 60% of anticancer drugs are natural products that have shown potential anticancer activities [2], such as anti-proliferation [3], anti-angiogenesis [4], reversal of multidrug resistance (MDR) [5], and antimetastasis [6] effects.

The genus *Aconitum* belongs to the family *Ranunculaceae*, which comprises about 400 species distributed in the temperate regions of the northern hemisphere, with half of them distributed in China [7]. Since ancient times, about 40 of these species have been widely used to treat apoplexy hemiplegia [8], asthma [9], and rheumatoid arthritis [10] in China, Japan, and other Asian countries; some examples are *A. carmichaeli* Debx., *A. kusnezoffii* Rchb., *A. sinomontanum* Nakai, and *A. leucostomum* Vorosch. [11]. Modern pharmacological studies have demonstrated that medicinal *Aconitum* plants can exert anti-inflammatory, analgesic [12,13], anti-arrhythmia [14], antioxidant [15], antibacterial [16], and anticancer effects [7,17]. In anticancer therapy, *Aconitum* plants have been proven effective for several types of cancer, such as lung, stomach, and liver cancers [18,19,20].

*Aconitum* plants chemically comprise alkaloids, flavonoids, steroids, and glycosides, and the main efficacy components as well as the toxic components are diterpenoid alkaloids [21,22,23], which are reportedly some of the most promising, naturally abundant compounds for treating cancer [24]. Diterpenoid alkaloids have been studied since the 1940s, and based on structural differences such as the number of carbon atoms on the mother nucleus, diterpenoid alkaloids are generally divided into four categories: C_18_-, C_19_-, C_20_-, and bis-diterpenoid alkaloids [25,26,27,28,29]. This review focuses on the progress of diterpenoid alkaloids with different structures derived from *Aconitum* plants and some of their derivatives (e.g., lappaconitine, aconitine, songorine, pseudokobusine, and 11-veratroylpseudokobusine) with potential anticancer activities. We also summarize some of their antitumor mechanisms. We hope this work can serve as a reference for further developing *Aconitum* diterpenoid alkaloids as anticancer agents.

## 2. Chemical Structure of Diterpenoid Alkaloids

Nearly a thousand natural diterpenoid alkaloids have been reported to date, and a large part of them originate from *Aconitum* plants [30], and C_19_-diterpenoid alkaloids are the most reported among them [27]. C_19_-diterpenoid alkaloids evolve from C_20_-diterpenoid alkaloids and degenerate into C_18_-diterpenoid alkaloids by losing the 18th carbon atom [25,31].

Based on the presence of oxygen-containing functional groups at the C-7 position, C_18_-diterpenoid alkaloids, which constitute a small group within the diterpenoid alkaloids, are classified aslappaconine or ranaconine types [28]. Concerning the carbon skeleton and substituents at specific positions, the C_19_-diterpenoid alkaloids may be initially divided into aconitine, lycoctonine, pyro, lactonepe, 7,17-seco, and rearranged types [27]. Compared with C_18_- and C_19_-diterpenoid alkaloids, the skeletal types of the C_20_-diterpenoid alkaloids are extremely complex, which may be divided into four classes, including 19 types [29]. The majority of C_20_-diterpenoid alkaloids have an exocyclic double bond structure and are generally divided into atisine, denudatine, hetidine, hetisine, napelline, and anopterine types nowadays [32].

**Figure 1:**
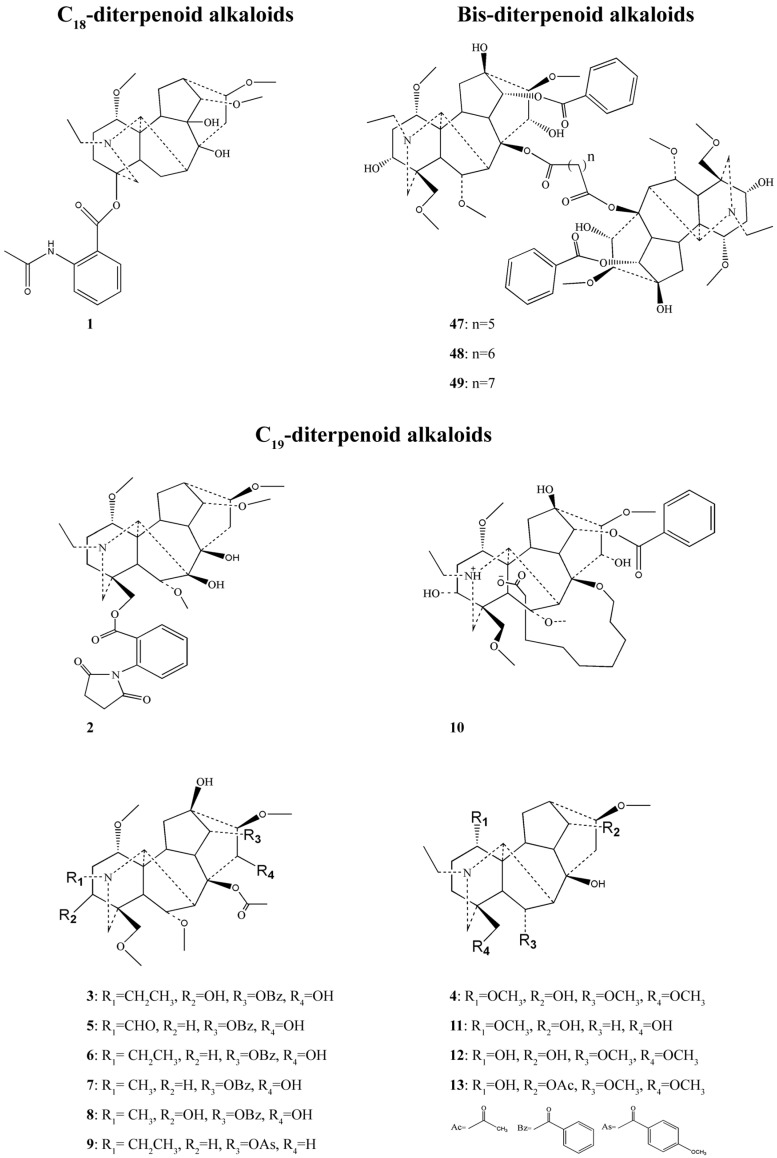
Chemical structures of the C_18_-, C_19_-, and bis-diterpenoid alkaloids with anticancer activities derived from the genus *Aconitum.*

**Figure 2:**
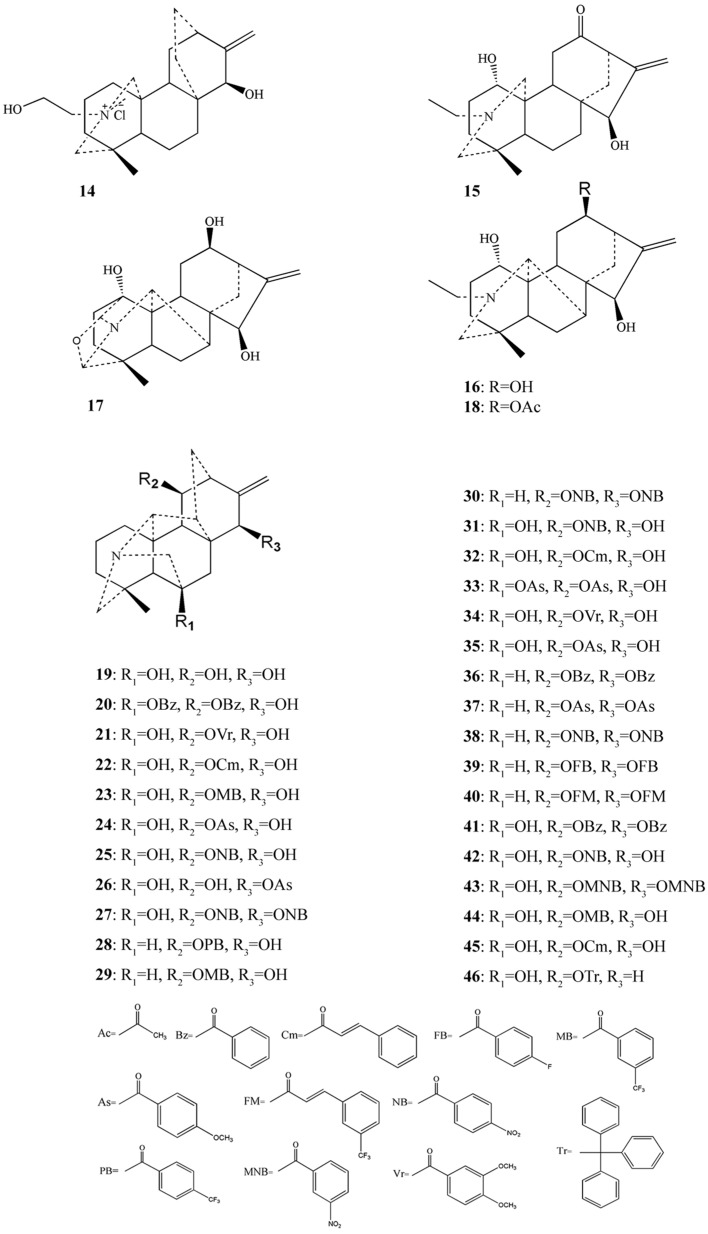
Chemical structures of the C_20_-diterpenoid alkaloids with anticancer activities derived from the genus *Aconitum*.

Figure 1 and Figure 2 show the chemical structures of C_18_-, C_19_-, bis-diterpenoid alkaloids and C_20_-diterpenoid alkaloids with anticancer activities derived from genus *Aconitum*, respectively.

## 3. Anticancer Activities of Diterpenoid Alkaloids

### 3.1. C_18_-Diterpenoid Alkaloids

Lappaconitine (**1**), a typical C_18_-diterpenoid alkaloid extracted for the first time in China from *A. sinomontanum* Nakai, is commonly used as postoperative analgesia and relief for clinical cancer pain as a non-addictive analgesic [33,34]. Lappaconitine exerts an analgesic effect by inhibiting the voltage-dependent sodium channels, increasing norepinephrine release in the synaptic cleft, and inhibiting the release of substance P [35]. Lappaconitine reportedly inhibits the proliferation of the human non-small cell lung cancer cells A549 dose dependently [36]. With increased lappaconitine concentration, the proportion of A549 cells increased gradually in G1 + G0 phase and decreased in S and G_2_+M phases, and the apoptosis rate increased with the down-regulated expression of Cyclin E1. Lappaconitine can also inhibit the expression of VEGF-A, and the combination of lappaconitine and oxaliplatin can arrest the cells in G1/G0 phase and inhibit the expression of Cyclin E1 [37].

As the derivative of lappaconitine (**1**), lappaconite hydrobromide can reportedly exert an efficient antitumor effect in mice by the National Institutes of Health (NIH) mice. In particular, the inhibition rates ranged within 11.20%–53.08%for liver tumor growth and within 29.81%–53.96% for S180 tumor growth [38].

### 3.2. C_19_-Diterpenoid Alkaloids

Lycaconitine (**2**) is a C_19_-diterpenoid alkaloid isolated from the roots of *Aconitum pseudo-laeve var. erectum* through bioassay-guided fractionation and repeated column chromatography. Although lycaconitine (**2**) does not present cytotoxicity to KB cells, it has potent inhibitory effects on pgp-MDR upon testing on the multidrug resistant human fibrocarcinoma KB V20C (resistant to 20 nM vincristine) [39].

In the 1980s, preliminary experimental studies on the antitumor effect of aconitine (**3**) were performed by multiple medical institutions. They demonstrated that 200 μg/mL aconitine inhibited the proliferation of gastric cancer cells by inhibiting its mitosis, and that the inhibitory rate of hepatocellular carcinoma in mice was 47.77%–57.38% [18]. Aconitine also reportedly has anticancer activity to the mice inoculated with gastric cancer cells and S180 cells, as well as the ability to inhibit the spontaneous metastasis of Lewis lung cancer cells [40]. Moreover, aconitine (150–400 μg/mL) can significantly inhibit the proliferation of Hepal-6 hepatoma cells in vitro, with the inhibitory rate of Hepal-6 cells in C75BL/6 male mice ranging within 26.12%–65.43% at concentrations of 0.15 and 0.375 mg/kg [41].

MDR is a key factor that hinders cancer treatment. The anticancer effect of aconitine (**3**) has been evaluated in drug-resistant human oral squamous cell carcinoma (KBv200), which shows that aconitine has a small inhibitory effect on the growth of KBv200 (IC_50_ = 224.91 μg/mL). However, aconitine can increase the sensitivity of vincristine to kill cells, and the IC_50_ values of vincristine in KBv200 are 0.2715 and 0.9185 μg/mL when combined with 12.5 and 6.25 μg/mL aconitine, respectively [42]. Thus, aconitine is considered to have no significant cytotoxic effect and can even reverse the MDR of cancer cells. Through immunohistochemistry and gene chip technology, follow-up studies has shown that aconitine can downregulate the expression of Protein Pgp and change the expression of Mdr1 gene by affecting apoptosis-related genes and the mitogen-activated protein kinase (MAPK) signal transduction system, thereby ultimately reversing the drug resistance [43,44].

(1α,6α,8α,14α,16α)-20-Ethyl-8,14-dihydroxy-1,6,16-trimethoxy-4-(methoxymethyl)-aconitane (**4**) was isolated from the roots of *Aconitum taipaicum* Hand.–Mazz, and cytotoxicity assays indicate that compound **4** exhibits stronger growth inhibitory than adriamycin against leukaemia cells HL-60 and K-562 [45]. In the same year, compound **4** has been found to inhibit the proliferation and invasion of HepG2 (liver hepatocellular carcinoma) cells and arrest cells in G0/G1 phase to promote cell apoptosis, the mechanism involves the upregulation of Bax and Caspase-3 expression and the downregulation of Bcl-2 (B-cell lymphoma-2) and CCND1 expression [46].

Found in *A. carmichaeli* Debx., five compounds including oxonitine (**5**), deoxyaconitine (**6**), hypaconitine (**7**), mesaconitine (**8**), and crassicauline A (**9**) show obvious cytotoxic activities against various cancers, such as leucocythemia, breast cancer, and liver cancer. Compared with two other diterpenoid alkaloids without cytotoxic activities, compounds **5**–**7** and **9** have two ester groups in the structure, which may have an effect on the cytotoxicity of the compounds [47]. 8-*O*-Azeloyl-14-benzoylaconine (**10**) is also a new C_19_-diterpenoid alkaloid with two ester groups in the structure found in the roots of *A. karacolicum* Rapcs. It shows good antiproliferative activities with an IC_50_ of about 10–20 µM against HCT-15 (colon cancer cell), A549 (lung cancer cell line), and MCF-7 (breast cancer cell line) cells [48]. 

Cammaconine (**11**) was isolated from the ethanol extract of *Aconitum vaginatum* Pritz. and identified by spectroscopic analysis. It has greater inhibitory effect on AGS (gastric cancer cell), HepG2, and A549 cells compared with 5-Fluorouracil [49]. Two C_19_-diterpenoid alkaloids, neoline (**12**) and 14-*O*-acetylneoline (**13**) were further isolated and identified from an enriched alkaloid fraction of *Aconitum flavum* Hand.–Mazz; they have been proven to possess growth-inhibition effects on human gastric carcinoma SGC-7901, hepatic carcinoma HepG2, and lung cancer A549 cells [50].

### 3.3. C_20_-Diterpenoid Alkaloids

Together with cammaconine (**11**), anatisine-type C_20_-diterpenoid alkaloid named atisinium chloride (**14**) was isolated from *A. vaginatum* Pritz. and found to inhibit the growth of various cancers [49]. In addition, songorine (**15**), 12-epi-napelline (**16**), and 12-epi-dehydronapelline (**17**) derived from *Aconitum flavum* Hand.–Mazz. inhibited the growth of SGC-7901 (gastric carcinoma), HepG2, and A549 cells such as neoline (**12**) [50].

In 2007, 13 natural diterpenoid alkaloids were isolated and purified from *Aconitum yesoense* var. *macroyesoense* and *Aconitum japonicum* and 22 derivatives were subsequently prepared from the parent alkaloids. The veatchine-type C_20_-diterpenoid alkaloid named 12-acetylluciculine (**18**) and the six derivatives designed from pseudokobusine (**19**), including 6,11-dibenzoylpseudokobusine (**20**), 11-veratroylpseudokobusine (**21**), 11-cinnamoylpseudokobusine (**22**), 11-(*m*-trifluoromethylbenzoyl)pseudokobusine (**23**), 11-anisoylpseudokobusine (**24**), and 11-*p*-nitrobenzoylpseudokobusine (**25**) are proven to inhibit the growth of human malignant A172 cells [51]. The hydroxyl groups at C-6 and C-15 of pseudokobusine are considered to be essential to the inhibitory effect, and the esterification of the hydroxyl group at C-11 may enhance such activity. In 2009, Koji Wada detected the anticancer activities of the same above mentioned diterpenoid alkaloids with four different cancer cells. They demonstrated that all six derivatives (**20**–**25**) have strong inhibitory activity against A172, A549, HeLa (cervical cancer cell line), and Raji (lymphoma cell line) cells (except compound **21** to HeLa cells) [52]. Compounds **23** and **24**, which show significant suppressive effects against Raji cells, have the same structure except for the group in the C-11 position. Compound **23** inhibits the phosphorylation of extracellular signal-regulated kinasein Raji cells but does not affect the growth of human CD34^+^ hematopoietic stem/progenitor cells, which can be significantly inhibited by compound **24** [53].

Ten new acylated alkaloid derivatives were prepared from the natural diterpenoid alkaloids of *A. yesoense* var. *macroyesoense* and *A. japonicum*; they are 11,15-dianisoylpseudokobusine (**26**), 11,15-di-*p*-nitrobenzoylpseudokobusin (**27**), 11-(*p*-trifluoromethylbenzoyl)kobusine (**28**), 11-(*m*-trifluoromethylbenzoyl)kobusine (**29**), 11,15-di-*p*-nitrobenzoylkobusine (**30**), 11-*p*-nitrobenzoylpseudokobusine (**31**), 11-cinnamoylpseudokobusine (**32**), 6,11-dianisoylpseudokobusine (**33**), 11-veratroylpseudokobusine (**34**), and 11-anisoylpseudokobusine (**35**). They inhibited the growth of A549 cells through G1 arrest, and their IC_50_ values ranged within 1.72–5.44 µM. Their cytotoxic effects can be enhanced by replacing an acyl group at both C-11 and C-15 positions [54].

In 2015, the antiproliferative effects of 108 diterpenoid alkaloids were tested by the same research team above against four cancer cells, namely, lung, prostate, nasopharyngeal, and vincristine-resistant nasopharyngeal (KB-VIN) cancer cell lines. The alkaloids that show substantial suppressive effects in 11 newly synthesized C_20_-diterpenoid alkaloid derivatives [55]: 11,15-dibenzoylkobusine (**36**), 11,15-dianisoylkobusine (**37**), 11,15-di-(4-nitrobenzoyl)kobusine (**38**), 11,15-di-(4-fluorobenzoyl)kobusine (**39**), 11,15-di-(3-trifluoromethylcinnamoyl)kobusine (**40**), 11,15-dibenzoylpseudokobusine (**41**), 11-(4-nitrobenzoyl)pseudokobusine (**42**), 11,15-di-(3-nitrobenzoyl)pseudokobusine (**43**), 11-(3-trifluoromethylbenzoyl)pseudokobusine (**44**), 11-cinnamoylpseudokobusine (**45**), and 11-tritylpseudokobusine (**46**). All of them were hetisine-type C_20_-diterpenoid alkaloids with two different substitution patterns of C-11 and C-11, 15, and the GI_50_s of them were summarized in Table 1.

### 3.4. Bis-Diterpenoid Alkaloids

Three bis-[*O*-(14-benzoylaconine-8-yl)]esters [56], including new semisynthetic alkaloids with diverse alkyl chains on the heterocyclic moiety, including bis-[*O*-(14-benzoylaconine-8-yl)]-pimelate (**47**), bis-[*O*-(14-benzoylaconine-8-yl)]-suberate (**48**), and bis-[*O*-(14-benzoylaconine-8-yl)]-azelate (**49**), built from the 8-*O*-azeloyl-14-benzoylaconine (**11**) skeleton, present remarkable cytotoxic activity in vitro against lung cancer A-549, colon cancer HCT-15, and breast cancer MCF-7 cells; their IC_50_s were <28 µM. The anticancer activities in vivo of bis-[*O*-(14-benzoylaconine-8-yl)]-suberate (**48**) was subsequently tested in immunodeficient mice transplanted with human tumors MCF-7 and HCT-15 cells because of its significant cytotoxicity in vitro. Its antitumor activity is obviously shown at a dose below the maximum tolerated dose. The impact of the alkyl-linker length of the designed bis-diterpenoid alkaloids on cytotoxicity is clearly elucidated in the study and can serve as a reference for designing novel antiproliferative agents [57].

## 4. Discussion and Conclusions

Diterpenoid alkaloids isolated and designed from *Aconitum* plants have shown effective anticancer properties in various cancer cell lines. Such properties include inhibiting cell growth, inducing apoptosis, interfering with the cell cycle, and altering MDR. The in vitro anticancer activities (IC_50_ values) of diterpenoid alkaloids derived from *Aconitum* and their derivatives are presented in Table 1. Some of them also exert noteworthy anticancer effects in animal models.

Most of natural diterpenoid alkaloids with anticancer effect in *Aconitum* are C_19_-diterpenoid alkaloids, although derivatives of C_20_-diterpenoid alkaloids also have notable anticancer potential. Many diterpenoid alkaloids tend to exhibit improved activity after simple structural modification [58], and many structures may affect the activity of a compound, such as the kind and position of substituents and the linker-chain length [59].

Diterpenoid alkaloids from *Aconitum* have great potential use as new drugs for treating cancer. This review can serve as a useful reference for researchers in their search for highly effective, low-toxicity diterpenoid alkaloids through structure modification and structure–activity analysis. We also provide a theoretical basis for safety medication in clinical settings and further development of new anticancer drugs.

## Figures and Tables

**Table 1:** The anti-proliferative activities of the diterpenoid alkaloids derived from the genus *Aconitum.*

No.	Compounds	Cancer Types	Cell Lines	IC_50_	Reference
**C_18_-diterpenoid alkaloids**					
**1**	Lappaconitine	Lung cancer	A549	6.71 × 10^3^ µM/48 h	[39]
**C_19_-diterpenoid alkaloids**					
**2**	Lycaconitine	Fibroblast carcinoma	KB V20C	110.65 µM/72 h	[39]
**3**	*Aconitine*	Oral squamous cell carcinoma	KBv200	348.29 µM/72 h	[42]
		Hepatoma carcinoma	Hepal-6	590.03 µM/48 h	[41]
		Hepatoma carcinoma	HePG2	0.85 × 10^−2^ µM/72 h	[47]
		Colon cancer	HCT8	8.12 × 10^−2^ µM/72 h	
		Breast cancer	MCF7	2.45 × 10^−2^ µM/72 h	
**4**	(1α,6α,8α,14α,16α)-20-ethyl-8,14-dihydroxy-1,6,16-trimethoxy-4-(methoxymethyl)-aconitane	Leukemia	HL-60	0.44 µM/24 h	[46]
		Leukemia	K-562	1.55 µM/24 h	
**5**	Oxonitine	Colon cancer	HCT8	29.48 × 10^−2^ µM/72 h	[47]
		Breast cancer	MCF7	3.13 × 10^−2^ µM/72 h	
		Hepatoma carcinoma	HePG2	8.61 × 10^−2^ µM/72 h	
**6**	Deoxyaconitine	Colon cancer	HCT8	5.14 × 10^−2^ µM/72 h	[47]
		Breast cancer	MCF7	10.35 × 10^−2^µM/72 h	
		Hepatoma carcinoma	HePG2	9.21 × 10^−2^ µM/72 h	
**7**	Hypaconitine	Colon cancer	HCT8	12.05 × 10^−2^ µM/72 h	[47]
		Breast cancer	MCF7	6.46 × 10^−2^ µM/72 h	
		Hepatoma carcinoma	HePG2	0.92 × 10^−2^ µM/72 h	
**8**	Mesaconitine	Colon cancer	HCT8	13.16 × 10^−2^ µM/72 h	[47]
		Breast cancer	MCF7	4.57 × 10^−2^ µM/72 h	
		Hepatoma carcinoma	HePG2	1.45 × 10^−2^ µM/72 h	
**9**	Crassicauline A	Colon cancer	HCT8	16.45 × 10^−2^ µM/72 h	[47]
		Breast cancer	MCF7	12.86 × 10^−2^ µM/72 h	
		Hepatoma carcinoma	HePG2	2.36 × 10^−2^ µM/72 h	
**10**	8-*O*-Azeloyl-14-benzoylaconine	Colon cancer	HCT-15	16.8 µM/24h	[48]
		Lung cancer	A549	19.4 µM/24 h	
		Breast cancer	MCF-7	10.3 µM/24 h	
**11**	Cammaconine	Gastric carcinoma	AGS	0.32 µM/48 h	[49]
		Hepatoma carcinoma	HepG2	34.55 µM/48 h	
		Lung cancer	A549	0.32 µM/48 h	
**12**	Neoline	Gastric carcinoma	SGC-7901	37.55 µM/48 h	[50]
		Hepatoma carcinoma	HepG2	28.36 µM/48 h	
		Lung cancer	A549	34.74 µM/48 h	
**13**	14-*O*-acetylneoline	Gastric carcinoma	SGC-7901	16.97 µM/48 h	[50]
		Hepatoma carcinoma	HepG2	33.76 µM/48 h	
		Lung cancer	A549	18.75 µM/48 h	
**C_20_-diterpenoid alkaloids**					
**14**	Atisinium chloride	Gastric carcinoma	AGS	0.44 µM/48 h	[49]
		Hepatoma carcinoma	HepG2	66.69 µM/48 h	
		Lung cancer	A549	2.29 µM/48 h	
**15**	Songorine	Gastric carcinoma	SGC-7901	46.55 µM/48 h	[50]
		Hepatoma carcinoma	HepG2	87.72 µM/48 h	
		Lung cancer	A549	61.90 µM/48 h	
**16**	12-epi-napelline	Gastric carcinoma	SGC-7901	64.79 µM/48 h	[50]
		Hepatoma carcinoma	HepG2	96.99 µM/48 h	
		Lung cancer	A549	65.91 µM/48 h	
**17**	12-epi-dehydronapelline	Gastric carcinoma	SGC-7901	65.00 µM/48 h	[50]
		Hepatoma carcinoma	HepG2	46.63 µM/48 h	
		Lung cancer	A549	76.50 µM/48 h	
**18**	12-acetylluciculine	Malignant glioma	A172	13.95 µM/24 h	[51]
**19**	Pseudokobusine	Malignant glioma	A172	>15.18 µM/24 h	[51]
**20**	6,11-dibenzoylpseudokobusine	Malignant glioma	A172	2.42 µM/24 h	[51]
**21**	11-veratroylpseudokobusine	Malignant glioma	A172	2.52 µM/24 h	[51]
		Lung cancer	A549	3.5 µM/24 h	[52]
**22**	11-cinnamoylpseudokobusine	Malignant glioma	A172	1.94 µM/24 h	[51]
		Lung cancer	A549	5.1 µM/24 h	[52]
**23**	11-(*m*-trifluoromethylbenzoyl)pseudokobusine	Malignant glioma	A172	Not shown	[51]
		Lung cancer	A549	4.4 µM/24 h	[52]
		Lung cancer	A549	4.67 µM/24 h	[54]
		Lymphoma	Raji	4.39 µM/96 h	[53]
**24**	11-anisoylpseudokobusine	Malignant glioma	A172	2.80 µM/24 h	[51]
		Lung cancer	A549	1.7 µM/24 h	[52]
		Lymphoma	Raji	5.18 µM/96 h	[53]
**25**	11-*p*-nitrobenzoylpseudokobusine	Malignant glioma	A172	3.13 µM/24 h	[51]
		Lung cancer	A549	3.5 µM/24 h	[52]
**26**	11,15-dianisoylpseudokobusine	Lung cancer	A549	1.72 µM/24 h	[54]
**27**	11,15-di-*p*-nitrobenzoylpseudokobusin	Lung cancer	A549	2.66 µM/24 h	[54]
**28**	11-(*p*-trifluoromethylbenzoyl)kobusine	Lung cancer	A549	5.44 µM/24 h	[54]
**29**	11-(*m*-trifluoromethylbenzoyl)kobusine	Lung cancer	A549	3.75 µM/24 h	[54]
**30**	11,15-di-*p*-nitrobenzoylkobusine	Lung cancer	A549	5.08 µM/24 h	[54]
**31**	11-*p*-nitrobenzoylpseudokobusine	Lung cancer	A549	4.24 µM/24 h	[54]
**32**	11-cinnamoylpseudokobusine	Lung cancer	A549	3.02 µM/24 h	[54]
**33**	6,11-dianisoylpseudokobusine	Lung cancer	A549	3.68 µM/24 h	[54]
**34**	11-veratroylpseudokobusine	Lung cancer	A549	4.07 µM/24 h	[54]
**35**	11-anisoylpseudokobusine	Lung cancer	A549	2.20 µM/24 h	[54]
**36**	11,15-dibenzoylkobusine	Lung cancer	A549	GI_50_ = 8.4 µM/72 h	[55]
		Prostate cancer	DU145	GI_50_ = 9.3 µM/72 h	
		Epidermoid carcinoma	KB	GI_50_ = 6.0 µM/72 h	
		Epidermoid carcinoma	KB-VIN	GI_50_ = 7.5 µM/72 h	
**37**	11,15-dianisoylkobusine	Lung cancer	A549	GI_50_ = 6.7 µM/72 h	[55]
		Prostate cancer	DU145	GI_50_ = 7.1 µM/72 h	
		Epidermoid carcinoma	KB	GI_50_ = 5.3 µM/72 h	
		Epidermoid carcinoma	KB-VIN	GI_50_ = 5.2 µM/72 h	
**38**	11,15-di-(4-nitrobenzoyl)kobusine	Lung cancer	A549	GI_50_ = 6.9 µM/72 h	[55]
		Prostate cancer	DU145	GI_50_ = 7.0 µM/72 h	
		Epidermoid carcinoma	KB	GI_50_ = 5.3 µM/72 h	
		Epidermoid carcinoma	KB-VIN	GI_50_ = 5.5 µM/72 h	
**39**	11,15-di-(4-fluorobenzoyl)kobusine	Lung cancer	A549	GI_50_ = 8.1 µM/72 h	[55]
		Prostate cancer	DU145	GI_50_ = 6.8 µM/72 h	
		Epidermoid carcinoma	KB	GI_50_ = 5.2 µM/72 h	
		Epidermoid carcinoma	KB-VIN	GI_50_ = 7.1 µM/72 h	
**40**	11,15-di-(3-trifluoromethylcinnamoyl)kobusine	Lung cancer	A549	GI_50_ = 5.5 µM/72 h	[55]
		Prostate cancer	DU145	GI_50_ = 6.2 µM/72 h	
		Epidermoid carcinoma	KB	GI_50_ = 4.1 µM/72 h	
		Epidermoid carcinoma	KB-VIN	GI_50_ = 3.1 µM/72 h	
**41**	11,15-dibenzoylpseudokobusine	Lung cancer	A549	GI_50_ = 8.8 µM/72 h	[55]
		Prostate cancer	DU145	GI_50_ = 7.6 µM/72 h	
		Epidermoid carcinoma	KB	GI_50_ = 5.2 µM/72 h	
		Epidermoid carcinoma	KB-VIN	GI_50_ = 6.3 µM/72 h	
**42**	11-(4-nitrobenzoyl)pseudokobusine	Lung cancer	A549	GI_50_ = 5.8 µM/72 h	[55]
		Prostate cancer	DU145	GI_50_ = 7.2 µM/72 h	
		Epidermoid carcinoma	KB	GI_50_ = 6.4 µM/72 h	
		Epidermoid carcinoma	KB-VIN	GI_50_ = 6.4 µM/72 h	
**43**	11,15-di-(3-nitrobenzoyl)pseudokobusine	Lung cancer	A549	GI_50_ = 5.0 µM/72 h	[55]
		Prostate cancer	DU145	GI_50_ = 5.2 µM/72 h	
		Epidermoid carcinoma	KB	GI_50_ = 5.6 µM/72 h	
		Epidermoid carcinoma	KB-VIN	GI_50_ = 5.6 µM/72 h	
**44**	11-(3-trifluoromethylbenzoyl)pseudokobusine	Lung cancer	A549	GI_50_ = 6.8 µM/72 h	[55]
		Prostate cancer	DU145	GI_50_ = 7.7 µM/72 h	
		Epidermoid carcinoma	KB	GI_50_ = 8.9 µM/72 h	
		Epidermoid carcinoma	KB-VIN	GI_50_ = 6.2 µM/72 h	
**45**	11-cinnamoylpseudokobusine	Lung cancer	A549	GI_50_ = 8.4 µM/72 h	[55]
		Prostate cancer	DU145	GI_50_ = 6.5 µM/72 h	
		Epidermoid carcinoma	KB	GI_50_ = 7.0 µM/72 h	
		Epidermoid carcinoma	KB-VIN	GI_50_ = 6.4 µM/72 h	
**46**	11-tritylpseudokobusine	Lung cancer	A549	GI_50_ = 6.4 µM/72 h	[55]
		Prostate cancer	DU145	GI_50_ = 6.0 µM/72 h	
		Epidermoid carcinoma	KB	GI_50_ = 6.6 µM/72 h	
		Epidermoid carcinoma	KB-VIN	GI_50_ = 5.3 µM/72 h	
**Bis-diterpenoid alkaloids**					
**47**	Bis-[*O*-(14-benzoylaconine-8-yl)]-pimelate	Lung cancer	A549	9.50 µM/72 h	[56]
		Breast cancer	MCF-7	7.56 µM/72 h	
		Colon cancer	HCT-15	4.64 µM/72 h	
**48**	Bis-[*O*-(14-benzoylaconine-8-yl)]-suberate	Lung cancer	A549	7.53 µM/72 h	[56]
		Breast cancer	MCF-7	6.90 µM/72 h	
		Colon cancer	HCT-15	4.01 µM/72 h	
**49**	Bis-[*O*-(14-benzoylaconine-8-yl)]-azelate	Lung cancer	A549	19.5 µM/72 h	[56]
		Breast cancer	MCF-7	16.9 µM/72 h	
		Colon cancer	HCT-15	28.0 µM/72 h	
